# Genetic assessment of serological and biochemical markers in Bharia tribe of Chhindwara district of Madhya Pradesh

**DOI:** 10.4103/0971-6866.73401

**Published:** 2010

**Authors:** Ruchira Chaudhary, Gunjan Sharma

**Affiliations:** Department of Zoology, Government Motilal Vigyan Mahavidhyalaya, Bhopal, Madhya Pradesh, India

**Keywords:** Bharia, Madhya Pradesh, sero-genetic

## Abstract

**BACKGROUND:**

The present sero-genetic study is the first of its kind to present the baseline data of Bharia tribe of Madhya Pradesh. The main aim of this study is to provide phenotype and allele-frequency data to characterize the population genetically and to fill the void on the genetic map of Madhya Pradesh.

**MATERIALS AND METHODS:**

For this, blood samples from 92 unrelated healthy individuals of Bharia tribe from Chhindwara district (Tamia block) were collected. Hemolysates prepared were analyzed for two serological (A1A2BO and Rh) and six biochemical (adenosine deaminase, adenylate kinase locus 1, acid phosphatase locus 1, phosphoglucomutase locus 1, esterase D and glucosephosphate isomerase) parameters, following the standard electrophoretic techniques.

**RESULTS:**

The Chi-square test for goodness of fit revealed no significant deviation between the observed and expected numbers in any of the seven genetic markers, suggesting that the tribe is in genetic equilibrium. A high incidence of B allele in A1A2BO blood group and low incidence of the A1 allele, with presence of A2 in only one individual, and a low frequency of Rh(D) (Rh negative allele) was observed in serological markers. Also, no rare variant was observed for biochemical markers.

**CONCLUSION:**

Principal Component Analysis done in order to detect the genetic affinity of Bharia tribe with other populations from the adjoining states of Madhya Pradesh based on the allele frequencies, showed a close association of Bharia with Gujarat and Rajasthan. Hence, this study has been helpful in revealing the genetic structure and affinity of Bharia tribe.

## Introduction

In the past three decades, a great deal of human genetic data has been generated, but not much work has been carried out on the serological and biochemical markers of the tribal population from the central Indian state of Madhya Pradesh. A large amount of data on ethnic, regional and social distribution of genetic markers has already been reported on Indian populations, but our knowledge about genetic variation of different genetic markers is still limited.[1–6] Although some studies have been carried out on the serological and biochemical markers,[7–9] only a small number of genetic data has been generated on the tribal population groups of Madhya Pradesh.[10–12]

Tribes are the assets of a country and the central Indian state of Madhya Pradesh is one of the largest states of India inhabiting the bulk of tribal population of the country (12,233,474), constituting 20.3% of the total population (60,348,023) of the state. Amongst a total of 46 scheduled tribes of the state, the main focus of this study was on Bharia tribe, mainly the inhabitants of the district Chhindwara (total population 20,890).[13] Bharia is listed as one of the primitive tribes of Madhya Pradesh. The word “Bharia” means roaming outside and it signifies their nomadic way of life in the past.[14] A small section of this community lives in Patalkot which is a bow-shaped formation on the Satpura plateau, an area consisting of ridges and valleys. The origin and genetic affiliation of Bharia tribe is still ambiguous, although there are several myths associated with it. According to one such belief, Bharias have descended from Bhar Kingdom, once dominant in eastern part of United Province (present Uttar Pradesh and Uttarakhand).[15] Occupationally, earlier they were *dhaya* (a system of shifting cultivation) cutters, but nowadays they rear cattle and sell milk and milk products. They are also farmers and mostly produce small millet called Kodo-Kutki. Other areas of activities include forest wealth collection and hunting.

The present study is therefore planned to present the serological (A_1_A_2_BO and Rh) and biochemical [erythrocyte enzymes adenosine deaminase (ADA), adenylate kinase (AK), esterase D (ESD), phosphoglucomutase (PGM), acid phosphatase (ACP) and glucose phosphate isomerase (GPI)] data of Bharia tribe from Chhindwara district of Madhya Pradesh.

## Materials and Methods

The present study comprises blood collection (about 1 ml each) from a total of 92 healthy unrelated individuals of either sex from Tamia block of Chhindwara district, after obtaining informed consent, in accordance with the ethical standards of Institutional Ethical Committee, following ICMR guidelines. Hemolysates, prepared by freezing and thawing technique, were electrophoresed for studying variabilities of different red cell enzymes in Bharia tribe. The electrophoretic typing of the six erythrocyte enzymes was performed following the original techniques for ACP locus 1 (ACP1),[16] PGM locus 1 (PGM1),[17] AK locus 1 (AK1),[18] ADA,[19] and ESD.[20] The samples of Bharia tribal population were also analyzed for phenotypes of the A1A2BO and Rh(D) blood groups, using standard tube method.

### Statistical analysis

From phenotype data obtained after electrophoresis, allele frequencies were calculated using the gene counting method.[21] Chi-square tests were applied to check deviations from the Hardy-Weinberg equilibrium in the studied population. Heterozygosity was calculated from the observed allele frequencies for the purpose of measuring the genetic variability in the studied population.[22] In order to estimate the extent of genetic affinity of Bharia, the allele frequency values were compared with the earlier reported data of various tribes and caste population groups from Madhya Pradesh[10–12,23] and adjoining states. Dendrogram was constructed and Principal Component Analysis (PCA) was performed using SPSS version 15 software.

## Results

**Figure 1:**
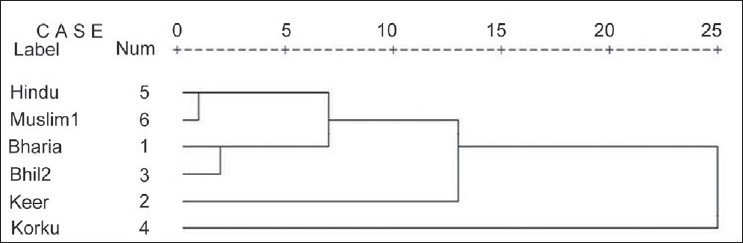
Dendrogram showing genetic relationship between Bharia and other tribes and castes of Madhya Pradesh.

**Figure 2:**
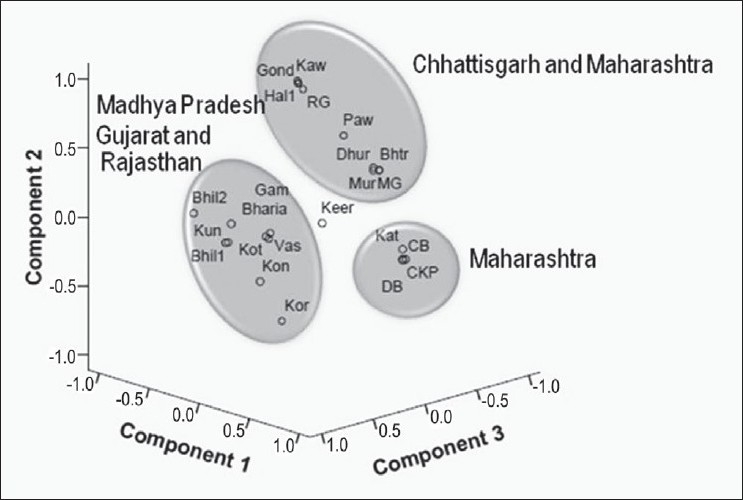
PCA plot based on allele frequencies of Bharia and other tribal and non-tribal populations of Madhya Pradesh and the adjoining states of Madhya Pradesh. Bhil1, Kor – Korku, Keer (Madhya Pradesh); Gond, Kaw – Kawar, Hal1 – Halba1, Dhur – Dhurwa, Bhtr – Bhatra, Mur – Muria, MG – MariaGond (Chhattisgarh); Paw – Pawara, Kat – Katkari, CB – Chitpavan Brahmin, DB – Deshastha Brahmin, CKP – Chandrayseny Kayastha Prabhu, RG – RajGond (Maharashtra); Kon – Konkanese, Gam – Gamit, Kun – Kunbi, Vas – Vasava, Kot – Kotwalia (Gujarat); Bhil2 (Rajasthan).

The phenotypic distribution of two serological markers (A1A2BO and Rh) and six erythrocyte enzymes (ADA, AK1, ESD, PGM1, ACP1 and GPI) are presented in Table 1 and their allele frequencies and locus-wise heterozygosity values are presented in Table 2. A dendrogram was constructed based on the allele frequency values of Bharia tribe in comparison with the published data of other populations of Madhya Pradesh [Figure 1], and on comparing the allele frequency with other populations from the adjoining states, a PCA plot was created using SPSS version 15.0 software [Figure 2].

**Table 1:** Phenotypic distribution of two serological and six erythrocyte enzyme systems in Bharia tribe

System	n	Phenotype	Observed	Expected	*χ*^2^	Statistical Significance
A1A2BO	92	O	25	26.87	3.14	*Non-significant (0.50> *P* >.30)
		A1	27	23.94		
		A2	1	1.08		
		B	33	30.48		
		A1B	5	9.16		
		A2B	1	0.495		
RH(D)	92	RH(D)+	92	–	–	–
		RH(D)−	0			
ADA	66	ADA 1	52	52.74	0.9253	†Non-significant (0.70 > *P* > 0.50)
		ADA 1,2	14	12.52		
		ADA 2	0	0.74		
AK1	90	AK1 1	78	77.47	0.6945	†Non-significant (0.80 > *P* > 0.70)
		AK1 1,2	11	12.06		
		AK1 2	1	0.47		
ESD	26	ESD 1	8	6.5	1.39	†Non-significant (0.70> *P* >.50)
		ESD 1,2	10	13		
		ESD 2	8	6.5		
PGM1	90	PGM1 1	42	40.005	0.904	†Non-significant (0.70> *P* >.50)
		PGM1 1,2	36	39.996		
		PGM1 2	12	9.999		
ACP1	24	ACP1 A	4	2.6661	1.5066	†Non-significant (0.50> *P* >.30)
		ACP1 A, B	8	10.6661		
		ACP1 B	12	10.6677		
GPI	91	GPI 1	90	–	–	–
		GPI 1,3	1			

* d.f. = 3

† d.f. = 2

**Table 2:** Allele frequencies and locus-wise heterozygosity of two serological and five erythrocyte enzyme systems in Bharia tribe

System	Allele	Allele frequency	Locus-wise Heterozygosity
A1A2BO	ABO*A1	0.1998	0.6059
	ABO*A2	0.0108	
	ABO*B	0.2491	
	ABO*O	0.5404	
RH(D)	RH*D	1	0
	RH*d	0	
ADA	ADA*1	0.8939	0.1897
	ADA*2	0.1061	
AK1	AK1*1	0.9278	0.1339
	AK1*2	0.0722	
ESD	ESD*1	0.5	0.5
	ESD*2	0.5	
PGM1	PGM1*1	0.6667	0.4444
	PGM1*2	0.3333	
ACP1	ACP1*A	0.3333	0.4444
	ACP1*B	0.6667	
GPI	GPI*1	0.9945	0.0109
	GPI*3	0.0055	
	Average Heterozygosity	0.2911

In the A1A2BO system, blood group B was found to be the most preponderant and A2 incidence was much lower, whereas the occurrence of A1 was observed to be more than that of O. In terms of allele frequency, highest frequency was that of B (0.2491) and lowest was of A2 (0.0108). In case of Rh system, Rh(D) was observed in all the samples of Bharia tribe. In each of the five enzyme systems considered, all the possible common phenotypes were found, but no example of any rare variant was encountered. For ADA, high allele frequency was recorded for ADA*1 (0.8939), which is found frequently in Indian population. Similar result was obtained for AK1 and PGM1 with high allele frequency of AK1*1 (0.9278) and PGM1*1 (0.6667), while for ACP1, allele ACP1*B showed high occurrence. Interestingly, ESD showed equal value for both the alleles, whereas ESD*1 is the most frequently occurring among the Indian population. Goodness of fit Chi-square test revealed that the distribution of phenotypes in each of the blood groups and enzyme systems typed was in genetic equilibrium in the studied Bharia tribe.

## Discussion

By and large, in the case of A1A2BO blood group system, it was observed that the average value of allele B was more as compared to allele A. Among the tribes, the value of B was high as compared to A; however, the difference between A and B frequencies was less among the tribes.[22] Similar values of allele frequency with minimal difference in values of A and B alleles were reported in Bhil tribe of Jhabua district, having a frequency of 0.221 in B allele and 0.219 in A1 allele.[10] On the contrary, Keer tribe and Hindu and Muslim caste population showed a major divergence in the frequencies of A and B alleles.[12,23] With regard to the Rh system, like other populations in the Indian subcontinent, it was characterized by relatively high frequencies of Rh(D) and absence of d allele. Similar case was observed in Dhurwa (Chhattisgarh), Rajgond and Katkari (Maharashtra) tribal populations.[5,24] As a general criteria, Rh(D) allele occurs at or less than a frequency of 1% in Asians. In other tribal populations of India, the frequency of Rh(D) shows a great deal of variation, but no definite trend has been observed in this case. Further, a low frequency of this allele in the tribes of Madhya Pradesh has been corroborated by earlier reports.[7]

As far as the erythrocyte enzyme markers are concerned, variations at different enzyme loci were observed. The present ADA*1 frequency in Bharia tribe was found similar to that observed in Keer,[12] while Bhil tribe from Jhabua illustrated a high value of ADA*1.[10] The ADA*2 allele frequency was found to be 10.61 in the present study. On a general account, the frequency of ADA*2 allele is quite frequent (~12%) in Indians.[25] The presence of high ADA*2 allele frequency in India suggests that perhaps this allele is of local origin, proliferating in the region on account of some selective advantage. For AK1 system, as observed in the present study, AK1*1 is the most frequent allele in all the populations studied, whereas the frequencies of AK1*2 allele are reported to be from 8 to 12%. In Bharias, the frequency of AK1*2 was around 7.2%, although low values have also been observed in Bhil tribe from Jhabua and Korku tribe from Hoshangabad.[10,11]

ESD showed remarkable equal frequency for both the alleles, ESD*1 and ESD*2, despite the fact that the frequency of common allele ESD*1 is reported to be 75% in the Indian population. Low sample size can be a possible reason for this, as earlier publications have shown an average allele frequency of 0.7315 for the other tribal populations of Madhya Pradesh.[10,12] ACP frequencies in Indian populations have a wide and interesting variation. In tribal populations, the ACP*B allele showed the highest frequency and the ACP*C allele was always low or nearly absent. Although the sample size was less in Bharia, nevertheless, the frequency of common allele ACP*B was found to be preponderant, with absence of ACP*C allele similar to other tribal populations of Madhya Pradesh.[10–12]

Both the PGM1*1 and PGM1*2 alleles are present in all populations in appreciable frequencies (63–80% and 20–37%, respectively) in Indians. No rare variant was observed in Bharia, while so far only Korku tribe from Hoshangabad have shown the occurrence of PGM1*7.[11] For GPI, unlike European or African populations, where GPI polymorphic variants are rare, most Indian populations have one or more variants. The common variant allele GPI*3 is frequently present in Indian populations.[26,27] In the present study, GPI*3 was found in a low frequency (0.005), similar to that of Bhil tribe from Jhabua (0.007),[10] whereas Korku tribe from Hoshangabad were monomorphic for this locus.[11]

Locus-by-locus comparison in Bharia suggests considerable intra-tribal variation in phenotype and allele frequencies. Chi-square value showed a good agreement between the observed and expected values in Bharia tribe. Both serological and biochemical markers were in genetic equilibrium as the differences between observed and expected frequencies were statistically not significant. Also, a considerable range of heterozygosity values across the loci was examined. The highest value of heterozygosity was found for the A1A2BO locus (0.6059) and the lowest for GPI (0.0109), with an overall mean of 0.291. On the other hand, PGM1 and ACP1 showed similar heterozygosity values (0.4444). The variation in average heterozygosity may simply be the genetic consequence of the population structure or it may indicate the effect of polymorphic or directional natural selection operating in the present Bharia tribal population of Madhya Pradesh. This wide variation in heterozygosity values from locus to locus also stresses the need for their estimation at a large number of loci to provide an adequate average heterozygosity estimate. Estimates of average heterozygosity ranging from 0.127 to 0.513 have been reported for Indian populations.[28,29] Some of the estimates are biased because only the systems known to be polymorphic and a small number of loci were taken into account.

The dendrogram [Figure 1] constructed based on the allele frequencies of Bharia and other tribal and non-tribal population of Madhya Pradesh illustrates that Bharia shows close affinity with the Bhil tribe from Jhabua district,[10] falling into one sub-cluster, whereas Hindus and Muslims from Indore district[23] form another sub-cluster, but adjacent to Bhil and Bharia tribes. On the other hand, Keer tribe from Sehore[12] and Korku tribe from Hoshangabad[11] appear to be different from the rest of the groups. The allele frequency of Bharia was compared with the allele frequencies of populations from other regions and the adjoining states of Madhya Pradesh, such as Maharashtra, Gujarat, Chhattisgarh and Rajasthan, in order to detect the genetic affinity of Bharia with other populations. On this basis, a PCA plot was constructed [Figure 2] which shows that Bharia forms a cluster with other tribal populations from Gujarat, whereas population from Chhattisgarh forms another cluster with the population of Maharashtra. On the other hand, the caste population from Maharashtra forms a separate cluster.

The present study has witnessed variation at different loci in the Bharia tribe, suggesting intra-population genetic differentiation. Every population is different from the other population in terms of its genetic constitution, but this diversity is more at the intra-population level than at the inter-population level. The present study has thus presented a possible illustration of the genetic structure, and the affinity of Bharia tribe of Madhya Pradesh has been revealed. Moreover, genetic studies on Bharia tribe featuring the variations at molecular level, which would help to further support the above presented data, are still under consideration.
